# Emulation of Sun-Induced Fluorescence from Radiance Data Recorded by the HyPlant Airborne Imaging Spectrometer

**DOI:** 10.3390/rs13214368

**Published:** 2021-10-29

**Authors:** Miguel Morata, Bastian Siegmann, Pablo Morcillo-Pallarés, Juan Pablo Rivera-Caicedo, Jochem Verrelst

**Affiliations:** 1 Image Processing Laboratory (IPL), Parc Científic, Universitat de València, 46980 Paterna, Spain; 2 Institute of Bio- and Geosciences, Plant Sciences (IBG-2), Forschungszentrum Jülich GmbH, 52428 Jülich, Germany; 3 Institute ITACA, Universitat Politècnica de València, 46022 Valencia, Spain; 4 CONACyT-UAN, Secretaría de Investigación y Posgrado, Universidad Autónoma de Nayarit, Ciudad de la Cultura Amado Nervo, Tepic 63155, Mexico

**Keywords:** emulation, machine learning, sun-induced fluorescence, sif, spectral fitting method (sfm), principal component analysis

## Abstract

The retrieval of sun-induced fluorescence (SIF) from hyperspectral radiance data grew to maturity with research activities around the FLuorescence EXplorer satellite mission FLEX, yet full-spectrum estimation methods such as the spectral fitting method (SFM) are computationally expensive. To bypass this computational load, this work aims to approximate the SFM-based SIF retrieval by means of statistical learning, i.e., *emulation*. While emulators emerged as fast surrogate models of simulators, the accuracy-speedup trade-offs are still to be analyzed when the emulation concept is applied to experimental data. We evaluated the possibility of approximating the SFM-like SIF output directly based on radiance data while minimizing the loss in precision as opposed to SFM-based SIF. To do so, we implemented a double principal component analysis (PCA) dimensionality reduction, i.e., in both input and output, to achieve emulation of multispectral SIF output based on hyperspectral radiance data. We then evaluated systematically: (1) multiple machine learning regression algorithms, (2) number of principal components, (3) number of training samples, and (4) quality of training samples. The best performing SIF emulator was then applied to a HyPlant flight line containing at sensor radiance information, and the results were compared to the SFM SIF map of the same flight line. The emulated SIF map was quasi-instantaneously generated, and a good agreement against the reference SFM map was obtained with a *R*^2^ of 0.88 and NRMSE of 3.77%. The SIF emulator was subsequently applied to 7 HyPlant flight lines to evaluate its robustness and portability, leading to a *R*^2^ between 0.68 and 0.95, and a NRMSE between 6.42% and 4.13%. Emulated SIF maps proved to be consistent while processing time was in the order of 3 min. In comparison, the original SFM needed approximately 78 min to complete the SIF processing. Our results suggest that emulation can be used to efficiently reduce computational loads of SIF retrieval methods.

## 1 Introduction

Essential indicators related to the plant’s actual health status can be derived by retrieving sun-induced fluorescence (SIF) from remotely sensed hyperspectral radiometric data, as seen in [1]. To this end, for the last few decades multiple SIF retrieval methods have been developed, most of them focusing on the retrieval of a single SIF estimate in specific absorption features such as Fraunhofer lines or in oxygen absorption regions (see [1–3] for reviews). More recently, full-spectrum methods such as the spectral fitting method (SFM) or PCA-based full-spectrum spectral fitting method (F-SFM) have been widely adopted to retrieve a range of values of the spectral signature in the range of the *O*_2_*A* and *O*_2_*B* absorption features coming from hyperspectral radiance data [4–7]. The main problem is that the SFM method applied to images is time-consuming because of the high computational demand of radiative transfer model simulations, the large number of per-pixel iterations, and the high number of pixels involved. For this reason, as for any other time-consuming processing step, there is a high demand for faster alternatives providing similar accuracy.

In this respect, with the purpose of bypassing the computational burden of intensive image processing steps, recently a computationally effective surrogate technique has been proposed by making use of machine learning regression algorithms (MLRAs) [8]. The aim of this work is approximating the original input–output relationships by a statistical learning model, which is less computational intensive, also referred to as a meta-model or *emulator* [9–12]. Emulation recently emerged as an acceleration technique in imaging spectroscopy applications such as synthetic scene generation [13] and in atmospheric correction routines [14,15]. The core idea is that, once trained, it is expected that the emulator allows generating SIF outputs quasi-instantaneously while high precision can be maintained. Pursuing this idea, in principle it should be possible to apply emulation to radiance-based SIF retrieval. Yet its feasibility and performances remain to be tested. For instance, among the challenges in developing such emulators, the question is how to deal with the hyperspectral nature of input, i.e., radiance data, and output data, i.e., multispectral SIF data. Earlier emulation studies dedicated to the generation of hyperspectral data suggested that this can be achieved with dimensionality reduction (DR) techniques, followed by the reconstruction of the output spectra [8,11–13,16]. Proceeding along this line, emulation has been mostly applied to achieve a fast input–output approximation of a deterministic radiative transfer model (e.g., [8,10–12,16]); however, when sufficient training data are available, emulators can be likewise developed based on experimental data, although it comes paired with some loss in accuracy. A first attempt in emulating experimental data was explored by [17], where a synthetic hyperspectral scene was emulated based on Sentinel-2 vegetation products. Nevertheless, the possibility of developing an emulator that converts one type of (hyper)spectral input data into another type of (hyper)spectral output data has not yet been explored. The concept can be appealing: if sufficient accuracy can be achieved, such emulators could serve as a computationally efficient image processing method to transform spectral data into a meaningful other spectral product.

Accordingly, with the purpose of offering a fast alternative for the rendering of SFM-like SIF maps, for this study, it was of interest to investigate the possibilities of emulating SFM-based SIF outputs in the range of the *O*_2_*A* absorption feature directly from hyperspectral radiance data. To fully exploit this proof of concept, this study evaluated multiple emulation strategies, i.e., analyzing the role of MLRAs, size and sampling strategy of training data, and tuning the dimensionality reduction step. Eventually, the optimized emulator is applied to radiance images that were recorded from the airborne imaging spectrometer HyPlant for fast generation of SIF maps. Validation against the reference SIF product formed the final step to verify the suitability of this acceleration technique.

The following sections starts with methods and materials. This section covers a theoretical framework, introduces the used experimental radiance and SIF data, the machine learning algorithms applied to build up emulators, and then is followed by the description of the experimental setup (Section 2). Section 3 provides the presentation of the achieved results. Finally, the main findings are discussed in Section 4 and Section 5 concludes the presented study.

## 2 Methods and Materials

### 2.1 Principles of Hyperspectral Data Emulation

Emulation is a statistical learning technique used to approximate model simulations when the model under investigation is too computationally costly to be run many times [9]. Emulators are based on machine learning and use a dataset made up of input–output pairs for training. In this way, the emulator is able to infer the statistical relationships on which the original complex model is based and thus imitate the behavior of the original model with a much lower computational cost. These data pairs should ideally cover the maximum multidimensional input space. Once the emulator is built, it is not necessary to perform any additional training of the model [9].

The challenge of emulating hyperspectral data, however, lies in predicting multiple spectral bands. Only a few MLRAs allow generating models that result in multiple outputs that represent the spectral profile. One approach to cover the large number of spectral bands involves to take advantage of the Hugens phenomenon, which shows the existence of spectral redundancy [18]. It implies that such data can be converted to a lower-dimensional space through DR techniques. Accordingly, spectroscopy data can be converted into components, which are only a fraction of the original amount of bands [16]. The classical principal component analysis (PCA) [19] is the most popular method and has shown its suitability to reconstruct satellite data and speed-up atmospheric RTMs [12,20–22]. Additionally, other DR methods can also be considered, such as partial least squares discriminant analysis [16].

When applied to spectral data, the first step in PCA consists of projecting the output spectra onto the first principle components (PCs), *p* ≪ *B*, where *p* is the number of selected components, and *B* is the number of original bands of the spectra. This is performed by obtaining the eigenvectors and eigenvalues of the estimated covariance matrix of the spectral inputs **X**. The eigenvectors matrix, **U**, is then used as a projection matrix that allows us to obtain the so-called **X**-scores, simply by W = **UX**. As **U** is an orthogonal matrix the reconstruction of **X** providing the scores is obtained by **X** = **U**⊤W. Hence, the spectra is reconstructed. By sorting the PCs from highest to lowest representation, we can vary the number *p* and evaluate the performance for PC. In this way, considering only a small number of PCs (low *p* value) will lead to a poor representation of the feature space and consequently model performance will be low. By increasing the number of PCs reconstruction of the feature space will improve, and at the same time, model performance increases. The goal is to find the optimal number of PCs that allows us to reduce the dimensionality of the input data (*p* ≪ *B*) while preserving the main spectral features. Thus, the MLRA first predicts the PCs, and afterwards the data are transformed back from the PCA domain (inverse PCA) to obtain the reconstructed output spectra at expenses of some loss in accuracy. Although this techniques requires iterative training, it is distinctly faster than train a MLRA with hyperspectral data without applying a DR. [5,8,12,16,23].

While DR methods are currently only applied to emulate hyperspectral output data based on a limited set of input variables (e.g., see [8,12,13,16]), in this work the challenge instead is the conversion of hyperspectral radiance input data into multispectral SIF output data. To enable such a spectrum-to-spectrum conversion, it is proposed to apply DR methods to both input and output data. Hence the same DR procedure as described above was applied to the input data only without applying the inverse PCA transformation. Thus, the developed emulator is based on a regression model that is calibrated with PCs of the hyperspectral input (radiance) and multispectral output variable (SIF). Finally, the output variable needs to be transformed back to the spectral feature space using an inverse PCA. This new strategy using PCAs to reduce the spectral dimension of the input and output variable of a regression model not only simplifies and accelerates the training phase, but also enables the development of an emulator, which has the capability to convert one type of (hyper)spectral data into another one.

**Figure 1:**
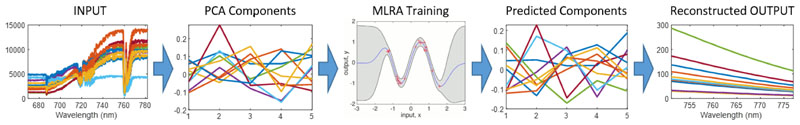
Diagram of the dimensionality reduction and reconstruction processing steps of the emulator.

A conceptual illustration of the developed emulation scheme is shown in Figure 1. The illustration starts with plotting some input hyperspectral data, here radiance data. In the example, we first applied a PCA to the input data and keep only the first 5 components. We also applied a PCA to the output spectral data, referred to as SIF bands, and again keep the first 5 components. We then trained the dataset with the input and output PC values to predict output PCs. Depending on the MLRA, two pathways are offered. For the single-output MLRAs the training phase loops over each output PC to train individuals models with the input PCs, thus leading to 5 models. Alternatively, some MLRAs have the capability of predicting multi-output, i.e., the model is trained only one time to produce all output PC at once. Finally, the predicted components were inverted to reconstruct the spectral output, i.e., SIF bands.

### 2.2 HyPlant Data and SIF Retrieval Using the Spectral Fitting Method

The SIF emulator concept was developed and tested using a dataset recorded by the airborne imaging spectrometer HyPlant. The HyPlant sensor system consists of the two modules DUAL and FLUO. In this, only the FLUO module was used, which has been specially designed to measure SIF. It covers the spectral range from 669.5–781.9 nm with a spectral sampling interval (SSI) of 0.11 nm, resulting in 1024 spectral bands [24].

The HyPlant FLUO dataset was recorded during the 2018 ESA FLEXSense campaign. The main aim of this FLEX preparatory campaign was to collect high-resolution airborne measurements of TOC reflectance and SIF across representative European monitoring sites located in Italy, Germany, France, Spain, and Switzerland, accompanied by ground-based measurements of important vegetation and atmospheric properties in parallel to Sentinel-3 satellite overpasses.

On the 26th of June 2018, HyPlant FLUO data of an area of approximately 15 × 10 km were acquired around the city of Jülich in the western part of Germany. The area is part of the Rur catchment and mainly consists of intensive agricultural fields and a large lignite open pit mine. In total, 7 flight lines, each of them having a spatial resolution of 3 m, were recorded from 1800 m above ground level.

The recorded HyPlant FLUO data were first converted from raw digital numbers to at-sensor radiance using coefficients provided by the sensor manufacturer and afterwards the SFM was applied to retrieve far-red SIF from the *O*_2_*A* absorption feature. While most SIF retrieval methods only determine a unique scalar value for the *O*_2_*A* absorption band, which will coincide with the maximum absorption wavelength (~760 nm) [25–28], the SFM provides SIF estimates for a specific spectral range around the *O*_2_*A* absorption feature.

The observed radiance at sensor level, is affected by atmospheric transmittance between the TOC target and the sensor. For this reason, the varying atmospheric optical path must be also included in the transmittance compensation strategy. However, these techniques cannot be extrapolated to airborne or satellite level, where the atmospheric path radiance (L_0_) and spherical albedo (S) must be included in the atmospheric correction scheme [29]. A combined surface–atmospheric radiative transfer model is used within the SFM approach to generate at-sensor radiance spectra around the *O*_2_*A* absorption band. The atmospheric spectra are computed by means of MODTRAN5 [30], in which the model input parameters are derived from sun photometer measurements (i.e., aerosol optical thickness (AOT) at 550 nm, water vapor column (WVC), and surface pressure) and geometry parameters derived from the navigation data of a HyPlant image cube. The Spectral Fitting uses general mathematical functions to model the canopy fluorescence and reflectance at different wavelengths within spectral window centered on the oxygen absorption feature (750–780 nm for the O2-A) [7]. With successful fitting, SIF and reflectance are then decoupled [6]. The resulting dataset provides information on SIF in 27 spectral bands covering the spectral range from 751-77 nm. A detailed description of the HyPlant FLUO processing scheme can be found in [7]. The PC used for SFM processing has the following characteristics: Windows 10 Pro 64-bit OS, Intel i7-6850K, 3.60 GHz, 32 GB RAM,using 6 real cores for the processing. With this computer, the SIF retrieved from HyPlant radiance data takes approximately 1.56 seconds for 1000 samples.

### 2.3 Machine Learning Algorithms for Emulation

The core machinery of an emulator is a trained machine learning regression algorithm (MLRA). A diversity of powerful MLRAs has been developed during the last few decades, and in numerous applications have proven their use of dealing efficiently with spectral data (see [17] for a review). MLRAs are well capable of capture the non-linear relationships that exist between datasets, and can therefore perfectly serve as candidates for developing an accurate emulator to convert one type of spectral data into another type. Based on experience in earlier emulation papers where MLRAs were systematically evaluated on their ability to reproduce spectral output [8,12,17], six advanced MLRAs were chosen that possess sufficient flexibility to convert hyperspectral radiance data into multispectral SIF data. They belong to the families of neural networks and kernel-based MLRAs. A brief description of the selected MLRAs is provided in Table 1.

### 2.4 Experimental Setup

Contrary to earlier emulation studies where the focus was on developing a surrogate model of a simulator, this study aimed to develop an emulator that converts experimental hyperspectral input data into multispectral output data for fast production of meaningful SIF output. The key steps of the pursued experimental setup are explained below.

The emulation and assessment experiments are based on relationships between spectral signatures of at-sensor radiance and the corresponding retrieved SIF spectral signatures in the spectral region of O_2_*A* absorption band. To obtain these training data, the HyPlant flight lines have been sampled by choosing random pixels distributed throughout the image, with the aim that the available spectral variability can be sufficiently captured. By training with experimental data, it is expected that the set of random values is representative of the entire variability of the complete input spectral space and, therefore, assures that the developed emulator will be able to reconstruct the correct spectral output for most spectral signatures. An important aspect hereby is that the training database size is known to influence the predictive power of the emulator [8,12], as a larger training dataset can capture more spectral heterogeneity. However, an increasing number of samples also results in longer training times and, to a lesser extent, runtime. At the same time, the database size can also cause problems due to random access memory (RAM) limitations in the training phase of the emulators. Given this all, in the analysis presented here, we built *training* databases with different numbers of samples to study the effect of database size on the accuracy, while keeping an acceptable compromise regarding the computation time and memory constrains.

The distribution of the training samples is another important aspect. When relying on image-based data, an undesirable consequence could be that in the training image one land cover type is highly represented covering a large part of the image, while another kind of surface is barely represented in this subset. This could cause an overfitting of the model to the training image. Moreover, when applying the emulator to other images, the land covers may not be equally distributed as in the training image. Spreading training pixels equal across land cover classes could provide robustness to the model and allow good performance on images that have not been used for training. Depending on the land cover heterogeneity in the image, a spatially uniformly distributed dataset will not necessarily be spectrally uniformly distributed. Consequently, in the case that in the training image a land cover is more spread than another, a spatially uniform distribution of the pixels would result in a feature space cluster distribution with a higher density of points in the areas corresponding to the dimension space of the land cover with the largest area covered in the image. In turn, there may be areas of the input dimensional space with a low sample density, corresponding to covers that spread small areas of the image. A possible solution to the problem at hand is to segment the image using an unsupervised classification method, and then to weight the training points based on the size of the area of the identified segments. Given these classes, three different sampling strategies have been followed, i.e., (1) random sampling without classification, and then segmented sampling according to (2) absolute number of pixels per class, and (3) with relative number of pixels per class. In the first strategy, the samples are distributed randomly in the image. In the second strategy, each class will have the same quantity of samples regardless of the area covered. In the third strategy, each class was represented by a number of samples proportional to the area it covers in the image. The segmentation is achieved by means of a k-means unsupervised classification method. K-means is a cluster method, which aims to partition a set of n observations into k groups in which each observation belongs to the group whose mean value is closest. The algorithm uses an iterative technique, given an initial set of k-random centroids, the algorithm assigns each point to the group with the closest mean and calculates the new centroids as the centroid of the observations in the group, these iterations are repeated until the algorithm converges to assignments that no longer change [38]. The number of classes of the two methods has been varied from 1 to 50 to analyze the effect of the number of classes on the the model accuracy.

**Figure 2:**
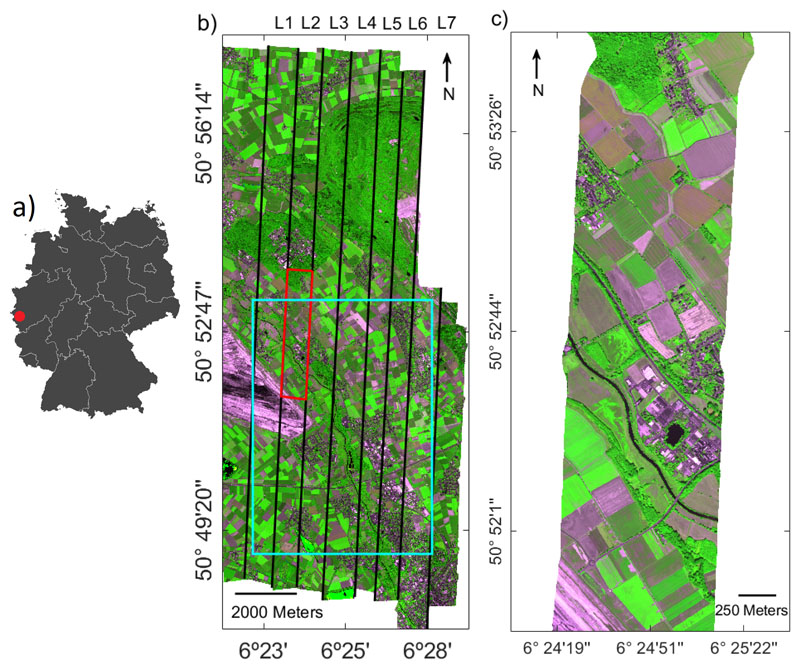
(**a**) Location of the study area in the western part of Germany. (**b**) RGB composite (700.1/754.4/674.4 nm) of the HyPlant FLUO mosaic consisting of seven flight lines (black framed areas) with the subset of flight line L2 used to build the emulator (red framed area). (**c**) Enlarged view of the subset of flight line L2.

The data used to train the emulator have been collected from a subset of a flight line (700 × 1500 pixels in size) that was acquired on 26 June 2018 at 13:46 (local time) from 1800 m above ground level (L2 Subset). The dataset used for the sampling scheme has been obtained from the training subset image (see Figure 2).This subset was favored given a great variety of land cover types, ranging from bare soil types, croplands, buildings, water to forest areas. Vegetation surfaces cover about the 61% of the image. Although non-vegetated surfaces do not have a SIF contribution, they can be useful to include in training to expand the feature space and so the robustness to the model. Given the abovedescribed sampling scheme, a sensitivity analysis has been conducted to find the optimal SIF emulator. Details about the investigated settings are given below.

1. The selected MLRAs have been evaluated using the training dataset. The default training settings were: 1000 random training samples, 20 PCs for the input, and 5 PCs for the output data.
2. PCAs were applied to the input and output data to reduce the feature space of both variables. To determine the optimal number of components, we varied the number of PCs in the input (from 1 to 50 PCs in steps of 5 while keeping the number of PCs in the output data constant at 5) and output data (from 1 to 10 PCs in steps of 1 while keeping the number of PCs in the input data constant at 20).
3. To investigate the effect of the number of samples on emulator performance we varied the number of samples from 200 to 7000 (200, 500, 700, 1000, 1500, 2000, 3000, 4000, 5000, 7000) while we fixed the number of PCs in the input and output data to 20 and 5, respectively.
4. The effect of the three different sampling strategies on emulator performance has been analyzed: (1) random sampling without classification, and segmented sampling according to (2) absolute number of pixels per class, and (3) relative number of pixels per class. Additionally, the impact of the number of classes used in unsupervised classification has also been tested by varying it from 1 to 50 classes (1, 2, 5, 10, 15, 20, 30, 40, 50).

**Figure 3:**
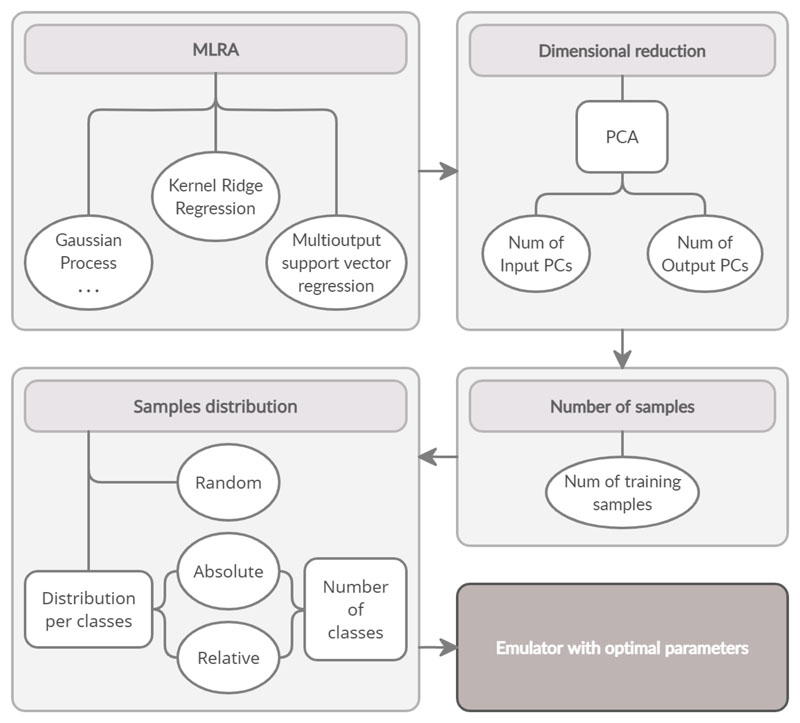
Flow chart showing the different parameters that were investigated to identify the optimal emulator. The grey boxes represent the analyzed parameters while the white ellipses indicate the different options tested for each parameter. The dark grey box represents the final emulator obtained with the optimal parameters of the analysis.

This systematic analysis allowed to identify the optimal emulator, which eventually enabled to accurately predict SIF from HyPlant FLUO data. The entire procedure is illustrated in the work flow in Figure 3. It summarizes the followed steps. First, multiple MLRAs were evaluated. The second step involved evaluation the effect of the number of PCs in input and output. In the third step, the size of the dataset was ranged to evaluate the effect in performance. Following, three sampling strategies were analyzed: random sampling and two kinds of segmented sampling with absolute and relative number of samples per class. Lastly, with the obtained optimal parameters, the final emulator was trained.

### 2.5 Emulation Validation

Since emulators are trained to mimic the original input–output relationship, they can only provide an approximation of this relationship and possibly introduce a source of uncertainty referred to as "code uncertainty" associated with the emulator [9]; therefore, an essential step prior to applying an emulator is to evaluate its prediction accuracy. First, a verification has been carried out as a function of wavelength to provide a first quick analysis of different MLRA emulator performance. To keep processing time low, similar to as in [11], a single random split was applied to the data using 70 % of the samples to train and the remaining 30 % to validate the emulator. The following goodness-of-fit statistics as a function of wavelength were calculated to evaluate the emulator performance. The root-mean-square-error (RMSE) and the normalized root-mean-square-error (NRMSE) were calculated per wavelength as:

(1) $$\text{RMSE}=\sqrt{\frac{1}{n}\sum_{i=1}^{n}{\left[\hat{f}\left(x_{i}\right)-f\left(x_{i}\right)\right]}^{2}},$$

(2) $$\text{NRMSE}=100\frac{RMSE}{f_{max}-f_{min}},$$

where *n* is the number of samples in the validation subset, $\hat{f}$ and *f*, respectively, the emulated and SFM reference SIF spectral values evaluated at the input point *x_i_*, and *f_max_* and *f_min_* are the maximum and minimum values of the *n* spectra in the reference dataset, respectively. Furthermore, in order to inspect the emulators accuracy and compare their performances along the spectral range, the spectral NRMSE_λ_ is also plotted as a function of wavelength.

Another statistic of the performance of the models used is the *R*^2^, which determines the linear correlation between the two measured variables (see Equation (3)):

(3) $$R^{2}=1-\frac{\sum_{i=1}^{n}{\left(f(x_{i})-\hat{f}(x_{i})\right)}^{2}}{\sum_{i=1}^{n}{\left(f(x_{i})-\bar{f}(x_{i})\right)}^{2\prime }}$$

where *n* is the number of samples in the validation subset, $\hat{f}$ and *f* are the emulated and SFM reference SIF spectral values evaluated at the input point *x_i_* and $\bar{f}$ is the mean of SFM reference SIF values, respectively.

### 2.6 Mapping Emulated SIF

The optimal emulator was first applied to the the same subset of flight line L2 (700 × 1500 pixels) that was used to sample the training pixels. A scatter plot providing information on the agreement of the emulated SIF map and the reference SFM SIF map at 760 nm together with the goodness-of-fit statistics were used to evaluate model performance. SIF at 760 nm was selected because the SIF community is probably more able to see the map at 760 nm where the oxygen absorption is at minimum. Additionally, a map providing information about the absolute error between both SIF maps was calculated to enable a more detailed analysis of the emulator’s prediction capability for the different land cover types. The optimal emulator was also applied to the entire flight line L2 to examine if the results are also reasonable for areas that were not included in the development of the emulator. Furthermore, the spatial transferability of the emulator was tested by applying it to the neighboring flight line L4 that covers a slightly different area and was recorded 16 minutes ealier. The same validation statistics were calculated to compare the emulated SIF map of flight line L4 to the corresponding reference SIF map retrieved with the SFM. Finally, the emulator performance was additionally evaluated for the entire area of interest by generating a SIF map based on the HyPlant FLUO mosaic consisting of seven flight lines.

### 2.7 Developed Software for Emulation Applications

The PC used for processing has the following characteristics: Windows 10 Enterprise v.19041.572 64-bits OS, Intel i7-9700K CPI 3.60 GHz, 32 GB RAM. All processing and evaluation steps were conducted within the in-house developed ARTMO (Automated Radiative Transfer Models Operator) software framework [39]. ARTMO is a scientific modular package developed in Matlab that provides tools and toolboxes for running a suite of leaf, canopy, and atmosphere RTMs and for post-processing applications such as the emulator toolbox [8]. As part of the ARTMO software package, the Emulator toolbox enables the evaluation of regression algorithms on their capability to approximate RTM outputs as a function of input variables [8,11]. Essentially, the emulator toolbox encompasses a suite of methods from the *simpleR* library [36] that can be combined with dimensionality reduction methods (e.g., PCA) in order to train statistical models that produce spectral outputs. While in earlier versions the focus of the Emulator toolbox was to train emulators based on RTM input variables to produce spectra as output, in its latest version (v.1.14) a new option is provided to insert a text file of (hyper)spectral data as input and output.

Moreover, in the here presented latest ARTMO version (v.3.28), the so-called new “LabelMe” tool has been introduced. This tool allows selecting pixels in one loaded image (e.g., radiance) and at the same time selects the corresponding pixels in a spatially related loaded image (e.g., SIF). Pixel selection can be performed manually, i.e., based on visual inspection, but also some automated selection options are provided, e.g., random or stratified sampling by means of k-means classification.

Finally, the selected samples can then be exported and used as LUTs to train our emulator models. The ARTMO toolboxes are freely downloadable at www.artmotoolbox.com, accessed on 29 October 2021.

## 3 Results

### 3.1 Analysis of SIF Emulation Strategies

**Figure 4:**
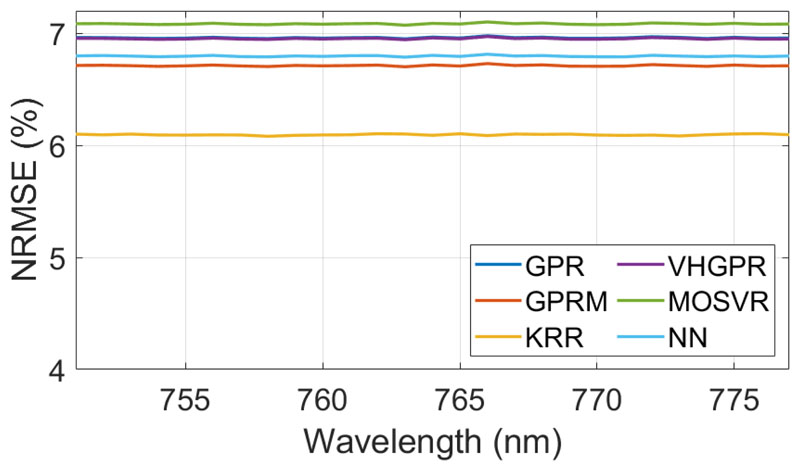
Spectral NRMSE (in %) results for the regression algorithms performance assessment as function of the five best regression algorithms, using (1000 samples, 20 PCA input, and 5 PCA output).

The predictive power of six MLRAs was evaluated to identify the optimal SIF emulator. The initial settings were: 1000 samples, 20 PCs for input, and 5 PCs for output data. A total of 70% of the collected samples were used to train the models while the remaining 30% were used for model validation. Validation results as well training time are provided in Table 2. The MLRA that led to the most accurate prediction of SIF was KRR, with a NRMSE lower than 6.1%. Additionally, KRR needed substantially less training time than any of the other algorithms. The accuracy of the different MLRAs for the investigated spectral range were calculated and are shown in Figure 4. Because the SIF signal in the *O*_2_*A* region is spectrally smooth, it results into a stable NRMSE over the 27 wavebands. Given KRR’s superior performance in both accuracy and speed to predict SIF, it was decided to continue with KRR in the further optimization steps.

Second, the influence of the DR method used to represent the input and output data was analyzed. Initially, PCAs with varying numbers of PCs were applied to the input dataset. The variation of computed NRMSE as a function of the number of PCs to the input data was analyzed to determine the optimal number of components that preserves most of the information content of the original radiance data. As expected, an increased number of PCs led to a more accurate emulator. The NRMSE continuously decreased, in the step from 10 to 15 the NRMSE decreases by 4.5% and in the step from 15 to 20 it decreases by 0.4%. From 20 PCs onwards the NRMSE variations are less than 0.6% reaching a stable value of approximately 6.7%. We therefore decided to use 20 PCs for the input data in further analysis.

**Figure 5:**
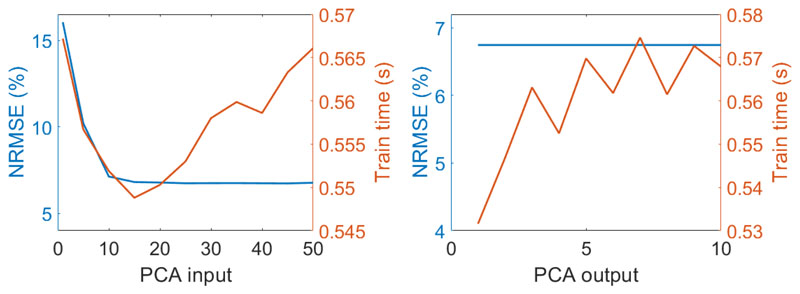
NRMSE (in %) (blue axis) and process time (orange axis) results for the KRR emulator performance assessment varying number of PCs in PCA input conversion (1000 samples, 5 PCA output) (**a**), and PCA output conversion (1000 samples, 20 PCA input) (**b**).

In case of the SIF output the NRMSE hardly varied along the wavebands. For all applied numbers of PCs the NRMSE was at a similar level of approximately 6.74% (Figure 5). This was not surprising since the 27 bands covering the *O*_2_*A* absorption feature are highly correlated Figure 1. Hence, we decided to use five PCs to represent the output data in further analysis. Since KRR is a multi-output algorithm, the model is only one time trained to deliver all the output PCs from the input PCs. It is important to note that although the training time normally increases with an increasing number of PCs, the time required for a higher number of PCs is not simply rising and sometimes behaves erratic. This is due to the fact that the processing time does not only depend on the used algorithm, but also on the uncontrollable internal processes of the used computer, which are not related to the required training time of the emulator.

**Figure 6:**
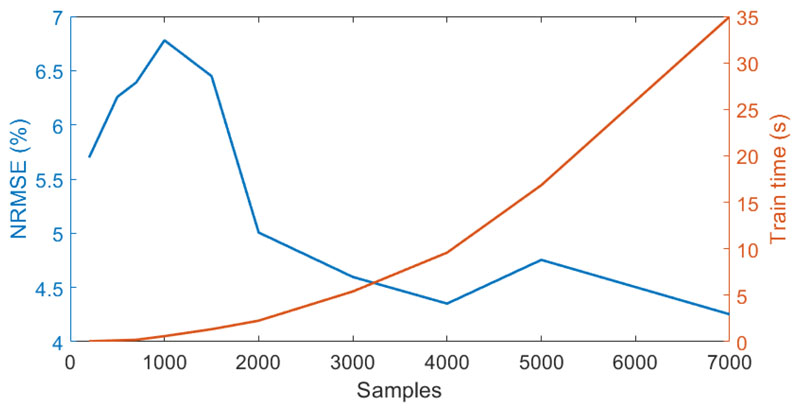
NRMSE (in %) (blue axis) and associated processing time (orange axis) of the KRR emulators (20 PCA input, 5 PCA output) built with a varying number of training samples.

Third, the effect of the training database size on the emulator’s performance was analyzed. Using the previously identified optimal number of 20 PCs for the input and 5 PCs for the output data the performance of the KRR model was studied by randomly increasing the number of training samples (Figure 6). Increasing the training sample size had the greatest effect on model performance. The NRMSE decreased from 7 to approximately 4% and leveled off when 3000 or more training samples were used. Adding more training samples, however, is accompanied by an increased time for model calibration. Hence, we strive to reach a reasonable trade-off between the number of samples and the associated time required for model training. Since the model trained with 3000 samples was identified as the best compromise of sample size and processing time, it was chosen to train the final emulator.

Under these premises, a study of the performance of the model has been conducted in three different sampling distributions: (1) ordinary random sampling, (2) forcing a relative sampling related to the land cover class area, and (3) absolute sampling per class. To evaluate the robustness of the sampling strategies, it must be ensured that model performance is similar for the training dataset and areas that were not included in model training (e.g., the full flight line). This provides information about the robustness of the emulator when applied to unknown data. The optimal settings (PCs input = 20, PCs output = 5, samples = 3000) were used to build emulators based on the subset of L2. The number of classes used within the three methods was varied to analyze the effect on model accuracy. The achieved results suggested that a variation of the class number within the different sampling strategies only has a low effect on model performance when 30% of the samples were used for validation. In case of using the entire flight line for model evaluation, the NRMSE leveled off at approximately 4% when using 40 classes for both sampling strategies.

We subsequently evaluated the robustness of the models based on the different sampling strategies when transferred to unknown image data. Emulators based on 40 classes were applied to the flight lines L3 and L6. Both flight lines were characterized by a different distribution of the land cover classes compared to flight line L2. The results are shown in Table 3. The results show that the model based on the absolute sampling provides the lowest NRMSE for both flight lines (L3 and L6).

Eventually the best evaluated emulator settings (20 PCs for the input data, 5 PCs for the output data, sample size = 3000, absolute sampling per class with 40 classes) were used in the further course of the study. For each parameter, the value from which leveled off the NMRSE was chosen, so higher values do not obtain an appreciable improvement moreover the computational cost increases substantially.

### 3.2 Application of the Emulator to a Subset of a Flight Line

**Figure 7:**
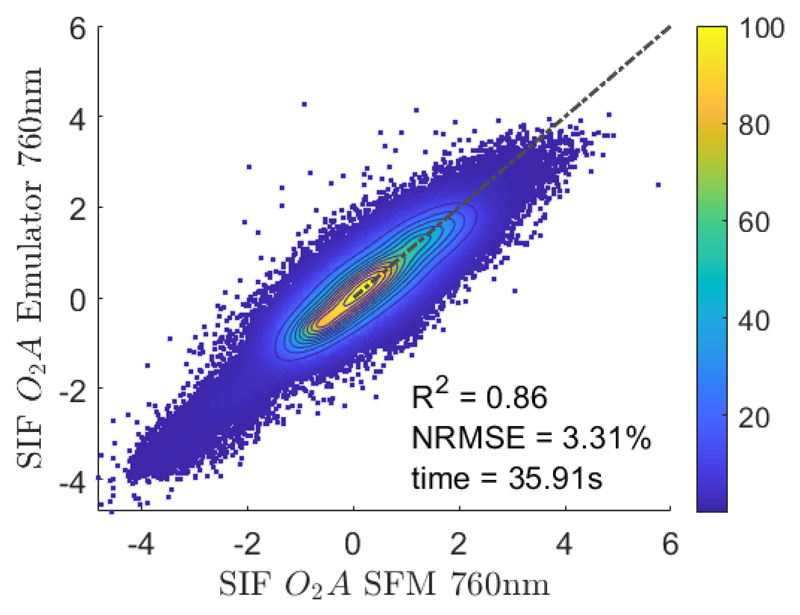
Scatter plot of the SFM SIF map retrieved at 760 nm and the corresponding map emulated with the developed KRR model for the subset of flight line L2. The x-axis represents the SIF values retrieved with the SFM while the y-axis shows the SIF values estimated by the emulator. The dashed line represents the 1:1-line. Units are in (mW m^−2^ sr^−1^ nm^−1^).

The trained emulator based on the optimal settings to estimate SIF from HyPlant FLUO at-sensor radiance data was applied to the subset of L2. The emulator allowed generating the *O*_2_*A* SIF product including all 27 spectral bands covering the spectral range from 751–777 nm with a spectral resolution of 1 nm. The emulated SIF map at 760 nm was then compared against the corresponding SFM-retrieved SIF map (Figure 7). The scatter plot illustrates the high agreement between the L2 SIF maps retrieved by SFM and the emulated SIF product. Most of the values are close to the one-to-one line (*R*^2^ = 0.86) and the scattering is relatively low (NRMSE = 3.31%).

**Figure 8:**
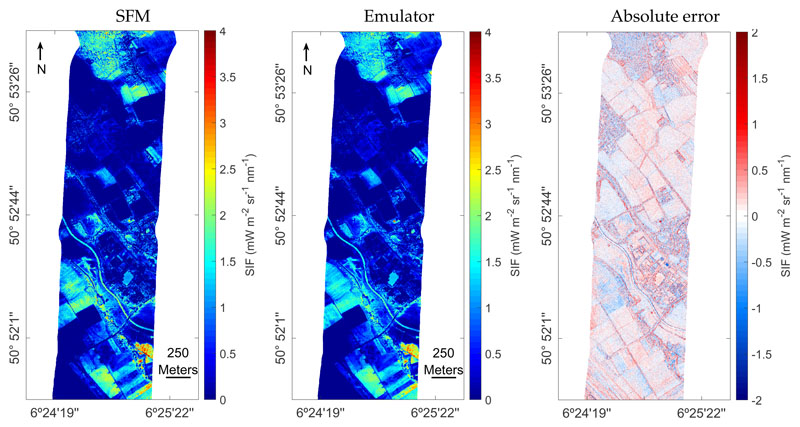
L2 SFM (**a**) and emulated SIF map at 760 nm (**b**) as well as the absolute error map (**c**) calculated as the difference of both maps.

**Figure 9:**
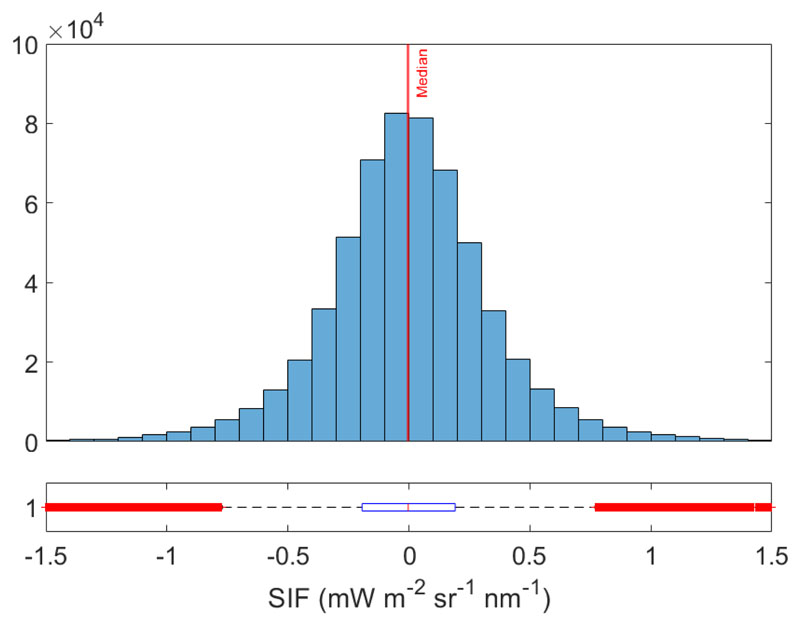
Histogram and boxplot of the absolute error. Lower and upper box boundaries are the 25th and 75th percentiles, respectively; red line medians are the lower and upper whiskers 2th and 98th percentiles, respectively; red crosses are data falling outside the whiskers.

To analyze the performance of the SIF emulator over different land cover types, both the SFM and emulated SIF maps and the absolute error between the SFM and emulated SIF map are shown in Figure 8. Both SIF maps show a similar spatial distribution of values and cover the same data range. The absolute errors are homogeneously distributed throughout the image, suggesting that the model was correctly adjusted to all the investigated classes. The absolute error map indicates a precise emulation of SIF for agricultural with few artifacts for small-scale heterogeneous areas such as forest or buildings where the shadows are more present. The absolute error distribution shows that the absolute error values are centered in a median of −0.003 and the 25th and 75th percentiles are in −0.19 and 0.19, respectively, see Figure 9.

### 3.3 Application of the SIF Emulator to an Entire Flight Line and Adjacent Flight Lines

**Figure 10:**
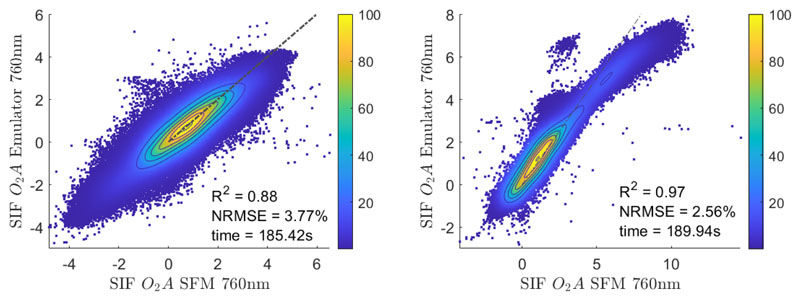
Scatter plot of the SFM SIF map retrieved at 760 nm and the corresponding map emulated with the developed KRR model for the entire flight line L2 (**left**) and flight line L4 (**right**). The x-axis represents the SIF values retrieved with the SFM while the y-axis shows the SIF values estimated by the emulator. The dashed line represents the 1:1-line. Units are in (mW m^−2^ sr^−1^ nm^−1^).

Subsequently, the SIF emulator was applied to the entire HyPlant FLUO flight line L2 to evaluate its performance for areas, which were not included in the training dataset. To validate the emulation result the same statistics as in the previous section were calculated by comparing the emulated SIF map to the reference SIF map retrieved with the SFM. Most of the values in the scatter plot in Figure 10 (left) show a high agreement and fit well to the 1:1-line. This is underlined by the high *R*^2^ of 0.88 and the low NRMSE of 3.77%. This consistency demonstrates the capability of the emulator to accurately predict SIF directly from at-sensor radiance data with low computational costs.

The SIF emulator was then applied to HyPlant FLUO image data of adjacent flight lines recorded close in time to the flight line L2 that the emulator was trained with. By doing this, it was possible to analyze the robustness and spatial transferability of the emulator. Figure 10 right shows the scatter plot of the SIF maps at 760 nm derived with the SFM and the emulated product. Overall, the SFM and corresponding emulated SIF values have a high level of agreement and show a good fit to the 1:1-line; however, the emulator slightly underestimates SIF values higher than 5 mW m^-2^ sr^-1^ nm^-1^. This underestimation is due to the fact that the model has been trained with pixel values collected from the subset of flight line L2, which only covered the data range between −5 and 6 mW m^−2^ sr^−1^ nm^−1^. Hence, it appeared that the emulator was not trained with very high SIF values and therefore the prediction of such values is uncertain. We can also observe a group of pixels for which the SFM provided values of around 4 mW m^−2^ sr^−1^ nm^−1^ and the SIF emulator estimated values of around 7 mW m^−2^ sr^−1^ nm^−1^. When inspecting the origin of these data, it appeared to be the sludge of a sewage plant, i.e., a land cover type that was not included in the training data. Except for a few small areas, the emulator was able to accurately estimate SIF, which is indicated by the very high *R*^2^ of 0.97 and the low NRMSE of 2.56%. These results underline the potential of the emulator to predict SIF from at-sensor radiance data of areas that were not included in model training.

### 3.4 Application of the SIF Emulator to All Flight Lines

When finally running the SIF emulator over all investigated flight lines it became clear that SIF of flight lines recorded before and after the training flight line are affected by over- and underestimated SIF values, respectively. The performance of the emulator strongly depends on the image acquisition time. The main reason for the over- and underestimation of SIF is that the image acquisition time directly affects the total radiance received by the sensor. Although data acquisition of the investigated flight lines was performed in a period of 13:06–14:01 in local time (LT), incoming radiation is continuously changing and even small differences have a strong impact on the recorded radiance and thus on the SIF signal. To overcome this problem, an improved SIF emulator was built with training data collected from three different flight lines (L1, L3, and L4) to take into account the variability in recorded radiance caused by the different time points of data acquisition for the single flight lines. This SIF emulator was built with 3000 samples, 1000 from each of the three flight lines, using the absolute sampling per class strategy. The different flight lines were recorded either in north (L1, L3) or south direction (L4) to ensure that the heading of the aircraft during data acquisition is also considered in the training of the emulator. Once the emulator was trained with the optimal parameters, it was applied to the entire set of flight lines and the results were evaluated by comparing them to the SFM maps in the same way as in the previous sections. Table 4 shows the quality statistics for the seven flight lines. Overall, the SIF emulation of the flight lines reveal a good agreement. However, L7 obtained a much lower *R*^2^ (0.68) than the other flight lines, which can be explained by the smaller size of the recorded area and the presence of buildings. As reported in the table, for all the emulated SIF maps *R*^2^ values of higher than 0.68 were achieved with the highest *R*^2^ of 0.95 for L4. The NRMSEs of all flight lines were lower than 6.5% with the lowest value of 4.13% obtained for L6. On average *R*^2^ for all flight lines is 0.84 and NRMSE 5.18%.

**Figure 11:**
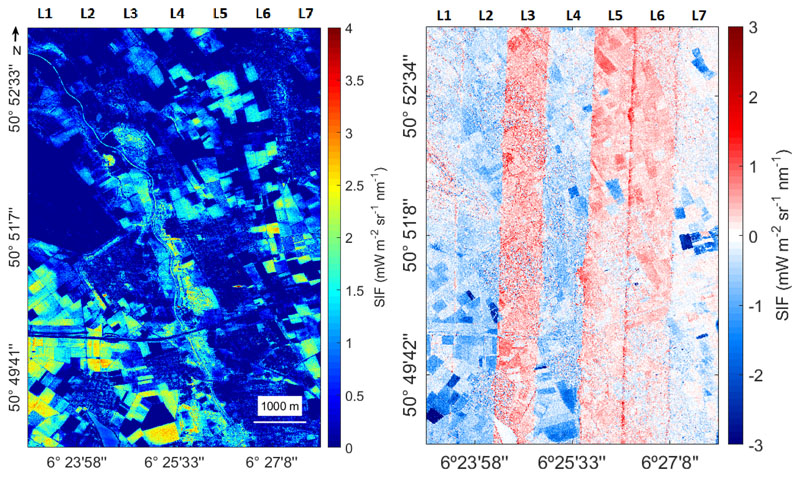
Subset of the emulated SIF mosaic at 760 nm using all investigated flight lines (**left**). Absolute error map calculated for the emulated and the SFM SIF mosaic at 760nm (**right**).

Lastly, a mosaic was made from the emulated SIF maps of all flight lines. A subset of the mosaic is presented in Figure 2. The optimal emulator was used to obtain the SIF value in the image (Figure 11, left) and an absolute error map was provided by comparing the reference image with the image obtained by means of the formulator (Figure 11, right). The emulated SIF map accurately captures the variability of crop values. By calculating the absolute error of the two images, a small bias for some flight lines can be observed, but in general, low absolute error values were obtained throughout the complete image.

## 4 Discussion

### 4.1 Interpreting SIF Emulator Results

Building upon previous studies where simulators were successfully approximated with statistical learning [8,10–12], here the concept of statistical learning was applied to emulate SIF at multiple wavelengths retrieved from HyPlant FLUO at-sensor radiance data. To optimize the predictive power of the developed emulator, key parameters were systematically analyzed, including: (1) different machine learning regression algorithms (MLRAs), (2) PCA as dimensionality reduction method with a varying number of components, (3) size of the training database, and (4) type of sampling scheme. The impact the different parameters had on the achieved results are discussed below.

The first analysis involved an accuracy assessment of several MLRAs. From the six investigated algorithms, KRR consistently achieved the highest accuracies. Additionally, KRR in comparison to other MLRAs has the advantage of low computational costs for both model training and application. On the one hand, KRR is characterized by a high efficiency, and on the other hand, it is relatively simple since only one hyperparameter needs to be optimized during model training. This excellent trade-off of good performance and low processing time KRR provides has previously been observed in other studies [8,13]. However, in earlier emulation studies that focused on approximating RTMs (e.g., PROSAIL, MODTRAN), GPR systematically outperformed KRR and other MLRAs [11,12,16] when simulated data were used for validation. These studies suggest that GPR is better suited for approximating deterministic models. In turn, when the data are more erratic, as the experimental data used in this study, KRR seems to be better suited, although the achieved accuracies were lower in comparison to deterministic models used to emulate RTM data. One reason for the superior performance of KRR in dealing with experimental data could be that only one hyperparameter needs be tuned, while GPR has three hyperparameters that potentially can be modified. KRR is therefore less able to specialize based on the training than GPR, likely leading to an advantage when applied to erratic validation data. Additionally, it must be noted that other popular MLRAs such as random forest and support vector regression have also been tested (results not shown). Both MLRAs did not reach the degree of accuracy as the methods presented in this study.

The second analysis focused on evaluating the DR method PCA and the number of PCs used to build emulators. DR is essential in the emulation of multi-input–multi-output data models, since it allows compressing spectral signatures with high spectral resolution. This greatly reduces the computational cost without losing information of the original data. We only analyzed the DR method PCA, which solves the multicolinearity problem in the model outputs without considering the correlations between dependent (outputs) and independent (inputs) variables. PCA is straightforward and allowed the fast training of emulators, even if a higher number of components was used. In principle, also alternative DR methods can be applied to improve the reconstruction of the output variables (e.g., [40]). For instance, partial least squares (PLS) [32] is another promising DR method, which takes into account the potential correlations of the input and output variables. PLS and PCA are mathematical identical in our scenario and in our case and results showed a similar but lower accuracy for PLS. For this reason, only PCA was analyzed in our study. In reality, we are not aware of other DR methods applied in other emulation studies. Since PCA was used as DR method in combination with KRR to develop emulators, the impact of the the number of PCs on model performance has been analyzed in detail. An increase in the number of components is expected to produce a more accurate representation of the initial spectral data and therefore a more accurate prediction model. Our results suggest that using more than 20 PCs to represent the input radiance data does not further reduce the error (NRMSE) of the model significantly. Hereby, it is well realized that this number depends on the complexity of the input data. As radiance data are characterized by specific absorption features, it is crucial to be sure that the number of PCs chosen are enough to represent the spectral variability. In comparison, typically about 20 components are sufficient for spectrally smoother reflectance data [11,41,42]. The same is true for the reduction and reconstruction of the SIF output data. SIF in the *O*_2_*A* region is spectrally smooth, implying that already one component captures the full variability. In fact, this would suggest that the emulator can be further simplified and accelerated by relying on one component, yet we preferred to stay with five components to ensure robustness and high speed.

A third analysis focused on evaluating the role of quantity and quality of the training database. Based on the usage of traditional interpolation methods, it was initially expected that larger training databases would lead to more accurate emulators. The results show that varying this parameter had the largest impact on model accuracy, leveling off from 3000 samples. One explanation is that adding more samples in the same input feature space does not add new information to the statistical model. With a few samples, the KRR emulator is able to mimic the SFM retrieval method to obtain the SIF spectrum from the radiance recorded by the sensor. It is also noteworthy that a higher number of samples in model training goes along with an increased processing time.

The last analysis was aimed to investigate the distribution of the training and validation samples (pixels) within the scene. Since the data used for model training and validation were composed of randomly chosen pixels from one flight line, the uniform distribution of samples in the input spatial space can lead to a non-uniform distribution in feature space and compromise the model accuracy. In the ideal case in which a model is trained with a large number of samples, the sample distribution assumed to be proportional to the area covered in the image by each of the land over classes. However, from a stochastic point of view, using a number of samples distinctly lower than the total number of pixels within the training image can lead to an unequal distribution of samples for the individual classes and thus to an over or under representation of single land cover classes characterized by a specific radiance and SIF signal. In the case of LUTs generated by RTMs, it is possible to evenly sample the entire input feature space, e.g., with the Latin hypercube sampling method [43], which is a commonly used sampling strategy for RTMs [16,44,45]. Additionally, alternative sampling strategies have proven to be more efficient than LHS. These might prove an important area for future research in emulation research [46,47]. However, if we obtain the training data from an experimental dataset such as an image, the spatial representativeness of the samples will be strongly determined by distribution and size of the land cover classes within the image. For instance, a spatially uniform distribution of the pixels would result in a cluster distribution with a higher density of points in the areas corresponding to the dimension space of the class with the largest area covered in the image; although, there may be areas of the input dimensional space with a low sample density, corresponding to land covers that are spread across small areas of the image. One way to overcome this problem is to classify the image first and then apply a random sampling in each land cover class separately. This will ensure a uniform distribution of samples so that each class is equally represented in the training dataset. For this reason, a segmentation was conducted to classify the image into various land cover classes to enable a stratified sampling with a similar number of samples in each class. As a third sampling strategy, pixels of each class were randomly selected proportional to the area the class covers in the training image, i.e., relative stratified sampling. From the three tested sampling strategies stratified sampling, which uses a fixed number of training samples per class, provided the best results, especially when the model was applied to unknown image data, which was not included model training.

MLRA models tend to optimize fitting to the training dataset, and thus provide high prediction accuracies for the entire image where training data were selected from [33]. However, if a model is applied to a dataset that was not included in the training phase, such as adjacent flight lines in this study that had slightly different acquisition times and were partly recorded by the aircraft flying in the opposite direction, model accuracies are distinctly lower. Such small differences can directly affect the total radiance received by the sensor. If an emulator is applied to images with different illumination geometries and irradiance intensities, it is important to include this variability by selecting training samples from all available image datasets to make the model more generally applicable. To overcome this problem, the final model was trained with samples collected from three flight lines, which were acquired at different times and had different data acquisition directions. Thus the emulator could adapt better to the different radiance intensities received by the sensor and provided higher SIF prediction accuracies.

In practice, each image may be acquired at different conditions and a bias may appear in the SIF output. (see Figure 11). Accordingly, to achieve a generally applicable and robust emulator, it is recommended to use a training dataset that includes multiple images taken with a broad range of characteristics [48]. In this study, airborne images acquired under clear sky conditions on a sunny day were used, so that atmospheric conditions only played a negligible role. Likewise, in future studies, emulators can be just as well trained based on radiance data coming from satellite images, e.g., as will be provided by FLEX [49]. The key is to include training data recorded at different geographic locations and under varying meteorological and atmospheric conditions. Altogether, regardless of what type of spectral data is to be emulated, one must always strive to make the emulator closely match with the images to be processed. While a sufficiently balanced training dataset is required to ensure production of accurate emulated output, at the same time, here also lies the weakness of the emulation technique, i.e., being dependent on training data. If the training data deviate too much from the image under study, the emulator will fail. One way to evaluate the success of an emulator over an image would be through per-pixel quantification of associated uncertainties. Although here KRR was evaluated as best performing both in accuracy and speed, when aiming to evaluate the quality of the emulator spatially, then GPR would be an attractive alternative. GPR, trained in a Bayesian framework, offers the advantage of delivering a standard deviation along with the mean estimate [50], so enabling inspecting the success of the emulated output map.

### 4.2 Opportunities for Emulation of Spectral Products

Emulators are increasingly used to approximate deterministic models of large computational burden. In this study, emulators are analyzed to convert one type of spectral data (here radiance) into another (here SIF). The final optimized emulator was able to generated SIF maps that proved to be consistent with SIF maps derived using the SFM approach. In terms of accuracy, the mean NRMSE was 5.18% in the O2*A* region. In terms of computational efficiency, the SIF emulation with KRR had a processing time of three minutes per flight line, while the SFM retrieval of the same images took around 78 minutes. The tremendous speedup in processing is fundamental in remote sensing image processing, where models face the challenge to process a massive amount of data at a high speed [12]. Finally, the memory consumption of an emulator is low, i.e., a KRR model is a few MBs in size, while RTM-based training data typically used in retrieval applications can rise to several GBs. Consequently, emulators can serve as an appealing alternative to a diversity of tedious remote sensing applications relying on RTMs. For instance, recent studies have demonstrated the efficiency of emulators in the context of atmospheric correction of remote sensing data [15,42], and here the potential of emulators to mimic the retrieval of SIF from at-sensor radiance was presented.

More generally, as the processing of optical remote sensing data from raw images to final products is a procedure that consists of several steps, some of those steps could be bypassed by emulators, i.e., radiometric correction: from digital numbers to radiometric data, and atmospheric correction: from radiometric data to reflectance data. Particularly, atmospheric correction can be time-consuming, and often requires specific software [51–54]. Alternatively, when properly trained, emulators can provide a fast approximation of the rigorous processing steps involved. Progress in this direction is expected e.g., with deep learning, with further acceleration and thereby improving the computational efficiency, accuracy, and physical awareness of the emulator [14]. Our work underlines the importance of different steps in model training to build a robust emulator, which is able to produce accurate SIF maps from radiance data of different flight lines recorded under changing illumination conditions. When aiming to further explore the potential of emulators for other fields of application in remote sensing, e.g., atmospheric correction, it is important to include a wide variability of scenarios under which the data were recorded, since even small changes in the external conditions can strongly affect the recorded spectral signal. From a practical perspective, we have expanded ARTMO’s emulator toolbox to enable such conversions, e.g., radiometric data to SIF or reflectance data. To analyze this proof of concept, follow-up studies are in preparation, e.g., to verify whether emulation can simplify the atmospheric correction step of the imaging spectroscopy sensor HyPlant, as well within the FLEX framework.

## 5 Conclusions

While the spectral fitting method (SFM) enables retrieval of multispectral SIF output in the *O*_2_*A* absorption band from TOA radiance data, the method is computationally costly given the many iterations involved. To bypass this computational burden, here we evaluated the idea of statistical learning, i.e., emulation, to approximate SFM-like multispectral SIF output coming directly from at-sensor radiance data. Experimental data came from the 2018 FLEXSense campaign where, from hyperspectral HyPlant acquisitions, the SFM method was applied for the retrieval of SIF outputs. Dimensionality reduction techniques were introduced to both input and output data to enable conversion of radiance to SIF data; these techniques have shown an excellent preservation of the original data quality. With the purpose of evaluating the accuracy that emulation can reach, multiple optimization strategies were systematically analyzed against a reference dataset, including the role of: (1) machine learning regression algorithms, (2) PCA dimensionality reduction, and (3) sampling strategy. Best accuracies were obtained with kernel ridge regression and a training dataset of 3000 samples, 20 PCs input, and 5 PCs output, with normalized RMSE of 4.16% This final emulator was subsequently applied to HyPlant flight lines to convert them into SIF output. It took a mere 3 minutes to process the 6x10^6^ pixels on a contemporary PC. SIF emulation accuracy were in the order of 5.18%, with the remark that flight line direction also influenced the emulation quality.

Summarizing, emulation offers a fast surrogate alternative to bypass the computational load of the SFM-based multispectral SIF retrieval method, although in the conducted experiments not all pixels of the HyPlant flight lines achieved a perfect reconstruction of SFM-like SIF output. When tolerating this trade-off in speedup at the cost of some loss in precision, the technique opens opportunities to convert any type of spectral data into another in a quick, computationally efficient way. Moreover, advances in machine learning and training strategies are expected to further improve the predictive power of emulation. To the benefit of the community, a GUI emulator toolbox has been developed that facilitates exploring further emulation applications.

## Figures and Tables

**Table 1:** List of selected MLRAs used for emulation implemented in ARTMO toolbox.

Algorithm	Brief Description	References
Neural Networks (NN)	NN are an interconnected group of nodes. Each node represents an artificial neuron with a connection from the output of one neuron to the input of another. Using the training dataset, weights are established for each neuron and the model is able to capture the non-linear relationships of the model. NN is multi-output.	[31]
Kernel ridge regression (KRR)	KRR minimizes the squared residuals in a higher dimensional feature space and can be considered as the kernel version of the regularized linear regression. KRR is multi-output.	[32,33]
Multioutput Support Vector Regression (MOSVR)	MOSVR extends the single-output SVR by taking into account the nonlinear relations between features but also among the output variables, which are typically inter-dependent. MOSVR is multi-output.	[34]
Gaussian process regression (GPR)	GPR is a nonparametric, Bayesian approach to regression. GPR has the ability to provide uncertainty measurements on the predictions. GPR is single-output.	[35,36]
Matlab Gaussian process regression (GPRM)	GPRM is similar to GPR but with the option to change multiple kernels https://es.mathworks.com/help/stats/kernel-covariance-function-options.html?lang=en, accessed on 29 October 2021. These kernels were initially tested, and the evaluated best trade-off between accuracy and speed was for “Squared Exponential”. GPRM is single-output.	[35]
Variational Heteroscedastic Gaussian Process Regression (VHGPR)	VHGPR is an anisotropic RBF kernel that has a scale, lengthscale per input feature, and a input-dependent noise power parameter as hyperparameters. VHGPR is single-output.	[37]

**Table 2:** Statistics obtained from the performance of the models used. RMSE is in (mW m^−2^ sr^−1^ nm^−1^).

MLRA	RMSE	NRMSE (%)	Time Train (s)
Kernel ridge Regression	0.30	6.09	0.57
Gaussian Processes Regression-Matlab	0.30	6.71	10.55
Neural Network	0.31	6.80	7.61
VH. Gaussian Processes Regression	0.31	6.95	80.14
Gaussian Processes Regression	0.31	6.96	23.80
Multioutput Support Vector Regression	0.32	7.08	12.33

**Table 3:** Determined model performance for unknown flight lines based on different sampling strategies. RMSE is provided in the unit of SIF (mW m^−2^ sr^−1^nm^−1^).

Sampling	Flight Line	RMSE	NRMSE (%)	*R* ^2^
Random	L3	1.14	8.16	0.81
	L6	0.98	5.72	0.87
Relative	L3	1.22	8.76	0.78
	L6	1.19	6.92	0.79
Absolute	L3	1.09	7.83	0.80
	L6	0.90	5.28	0.87

**Table 4:** Goodness-of-fit statistics obtained for the emulated SIF maps of all flight lines. The evaluation has been carried out by comparing the emulated SIF values with the corresponding values of SFM SIF maps on pixel basis.

	Acquisition Time (LT)	Direction	Num Pixels (Milions)	Processing Time (s)	RMSE (mW m^−2^ sr^−1^ nm^−1^)	NRMSE (%)	*R*_2_
L1	13:54	N	2.3	185.50	0.62	5.19	0.85
L2	13:46	S	2.3	182.93	0.73	6.42	0.82
L3	13:38	N	2.3	187.17	0.69	5.13	0.81
L4	13:30	S	2.3	185.87	0.81	5.19	0.95
L5	13:22	N	2.3	188.52	0.80	5.06	0.91
L6	13:14	S	2.3	186.25	0.68	4.13	0.88
L7	13:06	N	1.3	68.00	0.58	5.57	0.68

## Data Availability

Not applicable.
